# Population pharmacokinetics and exposure–response analyses of SAF-189s in Chinese patients with ALK+/ROS1+ non-small cell lung cancer

**DOI:** 10.3389/fphar.2024.1418549

**Published:** 2024-07-16

**Authors:** Yinhui Liu, Yan Tan, Lin Hu, Jinlong Li, Jiansong Yang, Lei Diao, Jin Yang

**Affiliations:** ^1^ Center of Drug Metabolism and Pharmacokinetics, China Pharmaceutical University, Nanjing, China; ^2^ Department of Pharmacometrics, Mosim Co., Ltd, Shanghai, China; ^3^ Shanghai Fosun Pharmaceutical Development Co., Ltd., Shanghai, China

**Keywords:** SAF-189s, population pharmacokinetics, exposure–response relationship, non-small cell lung cancer, ALK+, ROS1+

## Abstract

**Objective:**

SAF-189s is a potent ALK/ROS1 inhibitor that is currently in clinical development for treating advanced ALK+/ROS1+ non-small cell lung cancer (NSCLC). Comprehensive population pharmacokinetics (PopPK) and exposure–response models were developed to evaluate the efficacy and safety of SAF-189s by integrating data from two clinical studies.

**Methods:**

The PopPK model was developed using plasma concentration data collected from patients with ALK+/ROS1+ advanced NSCLC (n = 299) and healthy subjects (n = 24). The covariates (demographics, laboratory values, subject types, and concomitant medications) were evaluated to determine their potential influence on the between-patient variability in the pharmacokinetics of SAF-189s. Individual exposure values were then used to investigate the relationships with the efficacy endpoints (overall response rate (ORR), progression-free survival (PFS), and duration of response (DOR)) and key safety endpoints (adverse events of interest).

**Results:**

The final PopPK model of SAF-189s was described by a one-compartment model with delayed first-order absorption and time-dependent elimination by allowing the clearance to decrease stepwise over time. Age was included as a covariate for apparent clearance (CL/F), while prior anti-cancer therapy in ALK+ patients (ALKPOT) was included for apparent volume of distribution (V/F). There were no apparent exposure–response relationships for any of the efficacy endpoints at doses of 80–210 mg. The relationship between exposure and safety suggested that a higher steady-state exposure was associated with more frequent incidences of hyperglycemia and proteinuria; the 210-mg dose group was also less tolerated than the other low-dose groups.

**Conclusion:**

PopPK and exposure–response models were developed for SAF-189s, and their results demonstrate that SAF-189s exposures are at the plateau of exposure–response for efficacy. The 210-mg dose group had a significantly higher safety risk, while the 160-mg dose group was well-tolerated. Thus, 160 mg of SAF-189s once daily was selected as the recommended phase III dose for the ALK+/ROS1+ or ROS1+ NSCLC patients.

## 1 Introduction

Lung cancer is the leading cause of cancer-related mortality globally, with non-small cell lung cancer (NSCLC) constituting approximately 85% of all reported lung cancer cases (Tsvetkova and Goss, 2012; Ferlay et al., 2015). ALK and ROS1 are well-known mutated/rearranged oncogenes observed in patients with NSCLC, and the ALK rearrangements account for 2%–8%, while the ROS1 rearrangements for 1%–2% (Soda et al., 2007; Gainor and Shaw, 2013; Kris et al., 2014). This disease is characterized by high risk of central nervous system (CNS) metastases, especially a high prevalence of brain metastases at diagnosis (Rangachari et al., 2015; Economopoulou and Mountzios, 2016; Johung et al., 2016). Crizotinib was the historical standard for first-line treatment of ALK/ROS1 positive NSCLC. However, many patients treated with crizotinib would relapse within 1 year, primarily due to poor CNS penetration or development of resistant mutations (Costa et al., 2011; Chun et al., 2012; Awad and Shaw, 2014; Poon and Kelly, 2017).

SAF-189s is a potent oral ALK/ROS1 inhibitor with significant *in vitro* antitumor activity. Strong antitumor effects have been demonstrated in tumor xenograft models derived from genetically engineered cells overexpressing ALK rearrangements, ROS1+ rearrangements, or crizotinib-resistant ALK and ROS1 mutations, as well as in CNS tumor models (Xia et al., 2021).

A first-in-human phase I/II study (NCT04237805) enrolled patients with advanced ALK+/ROS1+ NSCLC with or without asymptomatic CNS metastases. Based on the safety, efficacy, and clinical pharmacology data from phases I and IIa of the study, a dosage of 160 mg once daily (QD) SAF-189s was selected as the recommended amount for phase II (Yang et al., 2020). In phase II, the overall response rates (ORRs) were comparable for the overall ALK+ patients and patients with brain metastases at 78.7% (95% confidence interval (CI): 71.2–84.9) and 74.6% (95% CI: 62.9–84.2), respectively. The ALK-inhibitor-naive patients had ORRs of 92.3% (95% CI: 85.4–96.6) compared to 65.4% (95% CI: 44.3–82.8) in the crizotinib-pretreated group (Yang et al., 2022a). The independent review committee (IRC)-assessed ORR was 94.1% (95% CI: 71.3–99.9) with a median progression-free survival (PFS) of 16.5 months for the ROS1-naive patients in phase IIa; in phase IIb, the IRC-assessed ORRs were 80.4% (45/56; 95% CI: 67.6–89.8) for the overall ROS1-naive patients and 85.7% (18/21; 95% CI: 63.7–97.0) for the brain metastases subgroup (Yang et al., 2022b).

In a phase I food effect study, high-fat meals had minimal effects on the pharmacokinetic (PK) profile of SAF-189s compared to the fasted state following a single dose of 160 mg (geometric mean ratio: C_max_ of 109.1%; area under the curve: AUC_0-t_ of 105.1%), and the T_max_ (median: 6 h) and t_½_ (mean: around 35 h) were unaffected (Qin et al., 2023). The semi-log mean concentration−time curves for SAF-189s administered orally at a dose of 160 mg in the fasted state and after a high-fat meal show monoexponential declines. The intersubject variabilities were similar under the fasted and fed conditions for C_max_ (23.0% vs. 19.7%), AUC_0-t_ (33.8% vs. 31.3%), and AUC_0-∞_ (34.6% vs. 32.8%) (Qin et al., 2023).

Optimizing the dosing regimen during clinical development is a crucial step based on consideration of the covariates that significantly influence PK exposure and relationships between exposure and safety/efficacy outcomes. Herein, we describe the development of a population pharmacokinetics (PopPK) model characterizing SAF-189s plasma PKs based on data from its phase I/II study in Chinese patients with ALK+/ROS1+ NSCLC as well as a food effects study in healthy Chinese volunteers. The potential effects of the covariates were assessed, including the demographic factors and renal/hepatic function markers. The PopPK model of SAF-189s was used to characterize the population PK profile, explore factors that affected the exposure levels, and simulate PK parameters for the exposure–response (E-R) analysis. The E-R relationships of both the clinical efficacy outcomes and selected adverse events (AEs) were evaluated subsequently to support dose selection for the pivotal phase III study for patients with NSCLC.

## 2 Methods

### 2.1 Study design and subjects

The population PK analysis included data from one phase I study of SAF-189s conducted with healthy subjects (study STL31147; n = 24) and one phase I/II study conducted with Chinese patients having ALK+/ROS1+ advanced NSCLC (study SAF001; n = 299). STL31147 was a single-center, randomized, open-label, two-period (fed and fasting), and two-sequence crossover study on healthy Chinese adults. The volunteers received SAF-189s at a dose of 160 mg orally once in each period, with a washout period of 14 days. Blood samples (3 mL) were collected after each SAF-189s administration at 0 (predose), 1, 2, 3, 4, 6, 8, 10, 12, 24, 48, 72, 120, and 168 h post dosing. SAF001 was a multicenter, open-label, and single-arm phase I/II study to evaluate the safety, tolerability, pharmacokinetics, and antitumor activity of SAF-189s in patients with advanced ALK+ NSCLC (phase I) and ALK+/ROS1+ NSCLC (phase II). In the dose escalation portion, the study treatment included a single-agent 3-day PK run-in period and a continuous treatment period. The dosing regimens were 20, 40, 80, 120, 160, and 210 mg QD for 21 days in 21-day cycles during the continuous treatment period. In phase II, the patients received 80, 120, 160, and 210 mg of SAF-189s (phase IIa) or 160 mg (phase IIb) QD over 21-day cycles. The details of the study design and PK blood samplings are given in Table 1.

**TABLE 1:** Summary of the clinical studies included in the PopPK and exposure–response analyses.

Clinical study	Study design and subject population	Dosage	Number of Subjects	Sampling time points	Modeling
STL31147	Single-center, open-label, randomized, two-period (fed and fasting), two-sequence, cross-over healthy subjects	SAF-189s 160 mg orally once in each period	24	Each period: predose; 1 h, 2 h, 3 h, 4 h, 6 h, 8 h, 10 h, 12 h, 24 h, 48 h, 72 h, 120 h, and 168 h post SAF-189s administration	PopPK
SAF001	Phase I/II: multicenter, open-label, single-arm. Phase 1: dose escalation (including PK run-in and subsequent cycles), patients with ALK+ advanced malignant solid tumors. Phase II: Part 1: patients with ROS+ and ALK + advanced NSCLC. Part 2: Cohort 1: patients who had not received systematic treatment or only received first-line non-ROS1-inhibitor treatment. Except for PK run-in phase (single dose), all patients should take SAF-189s orally once a day continuously with a treatment cycle of 21 days.	Phase I: dose escalation, SAF-189s 20, 40, 80, 120, 160, and 210 mg QD. Phase II Part 1: SAF-189s 80, 120, 160, and 210 mg QD. Part 2: SAF-189s 160 mg QD.	Phase 1: 45 Phase 2 Part 1: 198 Part 2: 56	Phase I PK run-in (single dose) • D1: Dose-escalation phase: predose (within 5 min before administration), 1 h, 2 h, 3 h, 4 h, 5 h, 6 h, 12 h, 24 h, and 48 h post dosing. Expansion phase: predose, 1 h, 3 h, 5 h, 7 h, 10 h, 24 h, and 48 h post dosing. Once a day for continuous use • C1D1: predose • C1D8: predose • C1D15: Dose-escalation phase: predose, 1 h, 2 h, 3 h, 4 h, 5 h, 6 h, 8 h, 12 h, and 24 h post SAF-189s administration. Expansion phase: predose, 1 h, 3 h, 5 h, 7 h, 10 h, and 24 h post SAF-189s administration. • C2D1: predose • C3D1: predose Phase II • C1D1–C5D1: predose the first dose for each cycle • C2D1: 2 h, 5 h, and 24 h post SAF-189s administration	PopPK and exposure–response

* P.O., orally. QD, Once every day

All studies were conducted in accordance with the Good Clinical Practice guidelines and Declaration of Helsinki. The protocols were approved by the independent ethics committee of each investigation site, and all subjects provided informed consent. The tumor responses were assessed by the IRC based on the Response Evaluation Criteria in Solid Tumors (RECIST) 1.1 conditions. The exposure–efficacy analyses of SAF-189s were conducted based on data from patients in the SAF001 study to explore the relationships between systemic exposure and efficacy endpoints, including the ORR, PFS, and duration of response (DOR). The exposure–safety analyses of SAF-189s were conducted based on data from patients in the SAF001 study to explore the relationships between the systemic exposure and safety endpoints, including the adverse events (AEs) of interest. In these studies, the SAF-189s plasma concentrations were analyzed using validated liquid chromatography tandem mass spectrometry within the assay range of 0.5–150 ng mL^–1^.

### 2.2 Model development

Dataset preparation and exploratory graphical analyses were performed using R (version 4.2.0) and SAS^®^ (version 9.4, Institute Inc., Cary, NC, United States). The PK data of SAF-189s were pooled and analyzed using a non-linear mixed-effects model. The PopPK analysis was conducted using NONMEM^®^ software (version 7.3, ICON, Hanover, MD, United States) with first-order conditional estimations with interactions (FOCE-I). Following graphical exploratory analysis, one- and two-compartment models were evaluated; several absorption models were then tested, including first-order, zero-order, sequential zero- and first-order, and parallel first-order models with and without a lag absorption time; based on the characteristics of the elimination phase of the concentration–time profiles of SAF-189s, the linear, non-linear, and time-dependent elimination models (Lau et al., 2019; Kratochwil et al., 2021) were investigated. Interindividual variance (IIV) was then described as a log-normal distribution, and the covariance between the IIV parameters was assessed. Residual variability was assessed by comparing the proportional, additive, and combined error models. Owing to the low proportion (<1%) of post-administration samples with concentrations below the quantification limit (BQL) of the assay, the BQL data were excluded from the PopPK model development. Model selection was based on the objective function value (OFV), goodness-of-fit (GOF) plot, and robustness of parameter estimates. The ETA shrinkage was also taken into consideration when evaluating the models.

### 2.3 Covariate analysis

The covariates were prespecified based on their physiological and clinical relevance. The potential covariates included age, sex, weight, and subject type, in addition to the albumin (ALB), alanine aminotransferase (ALT), alkaline phosphatase (ALP), serum creatinine (SCR), creatinine clearance (CLCR), total bilirubin (TBIL), and co-administration values. After identifying the base model, scatter plots of the selected parameter estimates and ETA values from the base model vs. potential covariates are used to suggest the potential covariates having significant impacts on the PKs. Then, the potential covariates were evaluated using the stepwise covariate model (SCM) process. Stepwise forward addition was used to add the covariates to the model individually when the decrease in the OFV exceeded 3.84 (*p* < 0.05). Each covariate was removed from the full covariate model individually by the backward elimination method, and the least significant covariate that did not cause an increase in the OFV in excess of 10.83 (*p* < 0.001) was dropped from the model. This process was repeated until all remaining covariates were significant when removed individually.

### 2.4 Final PopPK model evaluation

The final model was developed by evaluating the precisions of the parameters, GOF plots, and OFV. Moreover, prediction-corrected visual predictive check (pcVPC; 1,000 simulation replicates) was used to assess the final model, and the 90% CIs of the 5th, 50th, and 95th percentiles of the simulated concentrations were visually compared with the actual observed data. Meanwhile, non-parametric bootstrap resampling was adopted to evaluate the model stability and estimate the CIs for the model parameters by repeatedly fitting the final model to the bootstrapped replicates (n = 1,000) of the dataset.

### 2.5 E-R analysis

The exposure–efficacy analysis entails evaluation of the relationships between the individual SAF-189s exposures and efficacy endpoints. These efficacy endpoints include the ORR, PFS, DOR, and tumor size. In Eq. 1, the Stein-Fojo biexponential (SFBE) model was used to characterize the tumor shrinkage (mean shrinkage rate, KS) and tumor growth inhibition (mean regrowth rate, KG) parameters to compute the sum of longest lesion diameters (SOD).

$$SOD_{ij}=\;I\left(t\leq 0\right)\times \left(SOD0_{i}\times \exp\left(KG_{i}\times t_{ij}\right)\right)+I\left(t>0\right)\times \left(SOD0_{i}\times \left[\exp\left(-KS_{i}\times t_{ij}\right)+\exp\left(KG_{i}\times t_{ij}\right)-1\right]\right)+e_{ij}.$$ (1)

The individual *post hoc* PK parameters from the final PopPK model and the most prevalent dosage were used to calculate C_min,ss_ and AUC_ss_ at steady state. The exposure was assessed for its relationship with the ORR using a logistic regression model and with PFS/DOR using the Cox regression model. The individual model parameter estimates (KG_i_, KS_i_) were plotted *versus* C_min, ss_i_ to explore the relationships between exposure and tumor growth.

The exposure–safety relationships were characterized using the exposure metrics to the first occurrence of an event. The AEs of interest selected for analysis included hyperglycemia and proteinuria. The individual *post hoc* PK parameters from the final PopPK model and the first dosage were used to calculate the C_min,ss_, C_max,ss_, and AUC_ss_ at steady state, which were used as the exposure metrics. The exposure–safety analysis was performed using a logistic regression modeling approach.

## 3 Results

### 3.1 PopPK analysis

A total of 3,538 measurable SAF-189s plasma concentration values were acquired from 323 subjects (24 healthy subjects and 299 NSCLC patients). Of these, 329 (9.30%) BQL and 6 (0.17%) non-BQL samples prior to first dosing, 30 (0.85%) BQL samples after dosing, and six subjects without concentration samples were excluded from the PopPK analysis dataset. Consequently, a total of 3,173 (89.68%) samples from 317 subjects (24 healthy subjects and 293 patients) were included in the final PopPK analysis.

**FIGURE 1:**
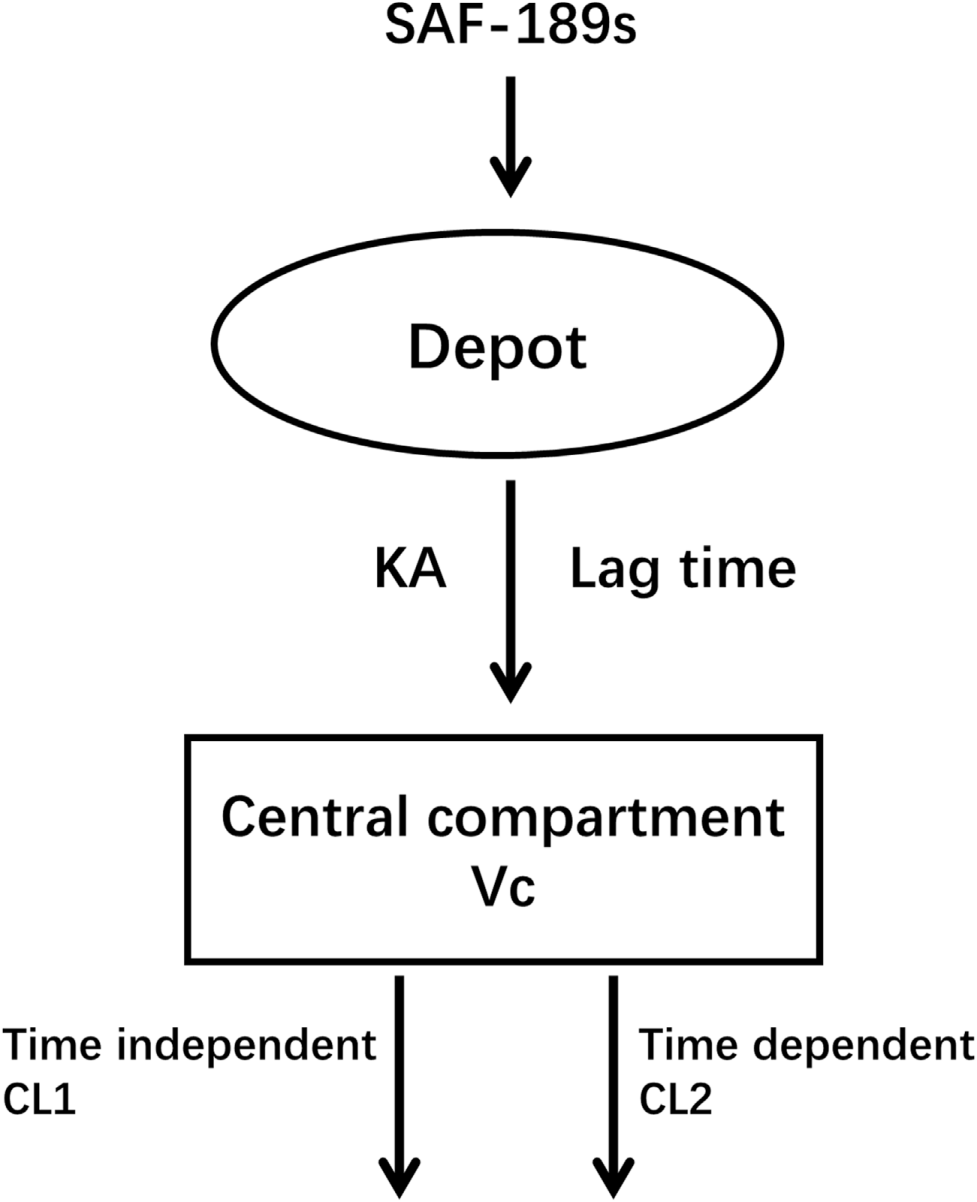
Base structure of the population pharmacokinetic model.

Of these 317 subjects, 272 subjects (85.8%) were <65 years of age (mean age 51.6 years, median age 53 years), and approximately half of them were women (56.5%). The average body weight was 63.2 kg (range: 37.3–92.5 kg), and average body mass index (BMI) was 23.7 kg m^–2^ (range: 15.5–34.8 kg m^–2^). A total of 41 (13%) subjects had mild hepatic impairment, and 94 (29.6%) and 1 (0.4%) patients had mild and moderate renal impairments, respectively. The other demographic and baseline characteristics of the PK-evaluable subjects are summarized in Supplementary document 1. The SAF-189s pharmacokinetics are well-characterized by the one-compartment model with oral first-order absorption based on the tlag time and time-varying inhibition of clearance (Figure 1).

In our effort to accurately describe the absorption delay of SAF-189s, we tested various transit compartment models. Despite these efforts, the pcVPC plots indicated that the transit compartment models did not offer significant improvements over the lag-time model. Considering both the predictive performance and computational efficiency, we selected the lag-time model as the final PopPK model; this model provided a satisfactory fit and allowed faster processing, thereby balancing accuracy and practicality in our analysis.

Since the PK data corresponded to samples collected after a single-dose administration and at steady state following autoinhibition, the apparent clearance (CL/F) of SAF-189s was estimated using the initial apparent clearance after single dosing (CL1/F) and subsequent time-varying apparent clearance at steady state (CL2/F).

$$CL_{i}=\left(TVCL1+TVCL2\cdot \exp\left(-Kout\cdot \left(DAY-1\right)\right)\right)\cdot \exp\left(\eta_{i}\right).$$ (2)

In Eq. 2, Kout is the time-varying inhibition rate constant, and DAY is the post-administration time. An exponential model was implemented to describe the IIVs, including CL1/F, CL2/F, apparent volume of distribution (V/F), KA, and tlag. Residual variability was described using a combined proportional and additive residual error model. Following the stepwise forward addition and backward elimination processes, the statistically significant covariates retained in the final model were age on CL and prior oncology therapy of ALK+ patients (ALKPOT) on V/F.

In the final PopPK model, the typical (±SE) values of CL1/F, CL2/F, V/F, KA, and tlag for SAF-189s were 64 (±1.51) L h^−1^, 40.3 (±6.89) L h^−1^, 5,210(±339) L, 0.501 (±0.0444) h^−1^, and 0.483 (±0.0306) h, respectively. Kout was estimated to be 1.35 (±0.421) day^−1^. For typical subjects (median age 53 years, ALK+ patients without previous ALK inhibitor treatment), the half-life was 34.6 h after the first dose; after multiple administrations to achieve steady state, the half-life was approximately 56.4 h. The exponent for the effect of age on CL was −0.314, suggesting faster clearance of SAF-189s in younger subjects. The V/F reductions were 26.6% in patients who received previous ALK inhibitor treatment (ALKPOT = 2, 3, 4), 8.3% in other patients (ROS1+ or unknown patients, ALKPOT = 5), and 21.6% in healthy subjects (ALKPOT = 6) relative to patients without previous ALK inhibitor treatment (ALKPOT = 1).

**FIGURE 2:**
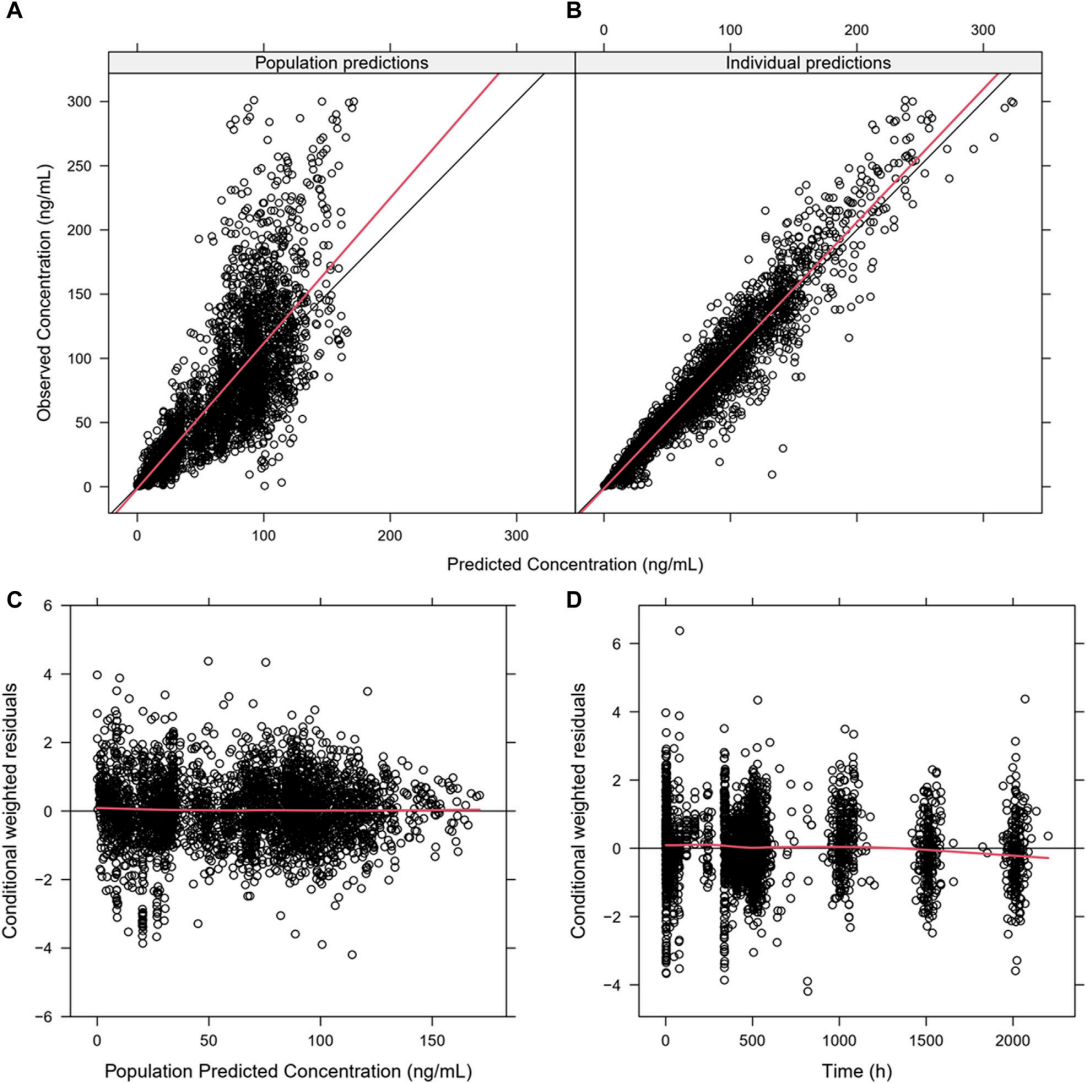
Goodness-of-fit plots for the final PopPK model. **(A)** Population predictions *versus* observations; **(B)** individual predictions *versus* observations; **(C)** conditional weighted residuals (CWRES) *versus* population predictions; **(D)** CWRES *versus* time. The plots show good correlations between the individual predictions and observation records. The CWRES had no misspecification of residuals related to population predictions, were mostly distributed within ±2, and well distributed along the zero line relative to population predictions.

**FIGURE 3:**
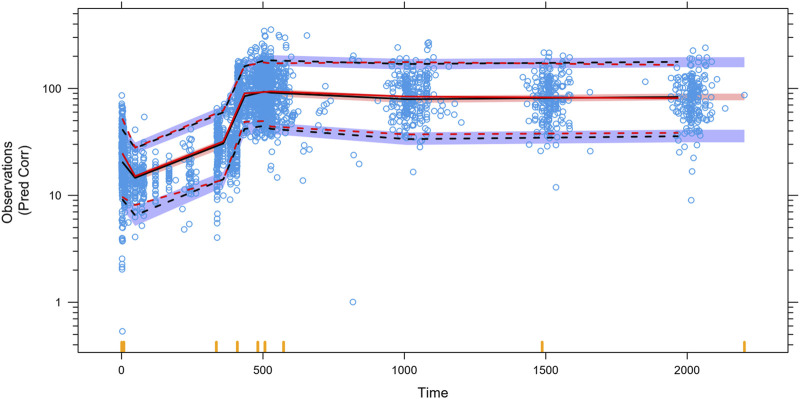
Prediction-corrected visual predictive checks (pcVPCs) for the final model. The blue open circles represent the individual observed concentrations. The red solid line and red dashed lines represent the median as well as 2.5th and 97.5th percentiles of the observed concentrations, respectively. The shaded area is the 95% confidence interval for the median (black) and the 2.5th and 97.5th percentiles of the results simulated 1,000 times from the final PK model. The 5th, 50th, and 95th percentiles of the observed concentrations were generally within the 90% prediction intervals.

Almost all PopPK parameters with relative standard error (RSE) values were less than 30%. The shrinkages of CL/F and V/F were each less than 30% (Table 2). The GOF diagnostic plots for the final model indicate excellent consistency between the observed and predicted data, as shown in Figure 2. A total of 1,000 bootstrap replicates were constructed, of which 884 were minimized successfully. The median values of the non-parametric bootstrap estimates of the parameters were consistent with the typical PK parameters of the final model. The pcVPC results are shown in Figure 3, where the model sufficiently reflects the data, and the 5th, 50th, and 95th percentiles of the observed concentrations are generally within the 90% CIs, indicating that the final model had a good description of the PKs of SAF-189s.

**TABLE 2:** Parameters of SAF-189s in the final PopPK model.

Final model						
Typical value					Bootstrap	
Theta	Description (Units)	Estimate	SE	RSE (%)	Estimate	95% CI
θ1	V/F (L)	5,210	339	6.5%	5,199	4,415–7,373
θ2	KA (L h^–1^)	0.501	0.0444	8.9%	0.504	0.445–0.585
θ3	CL1/F (L h^–1^)	64	1.51	2.4%	64	61.1–66.9
θ4	CL2/F (L h^–1^)	40.3	6.89	17.1%	40.5	25.5–57.5
θ5	Kout (L d^–1^)	1.35	0.421	31.2%	1.35	0.927–2.24
θ6	ALAG1 (h)	0.483	0.0306	6.3%	0.491	0.374–0.562
θ7	Age on CL/F	−0.314	0.0799	25.4%	−0.314	−0.476 to −0.167
θ8	ALKPOT = 2,3,4 on V/F	0.734	0.0649	8.8%	0.724	0.541–0.89
θ9	ALKPOT = 5 on V/F	0.917	0.0841	9.2%	0.905	0.729–1.12
θ10	ALKPOT = 6 on V/F	0.784	0.148	18.9%	0.782	0.557–0.939

Interindividual variability						
Eta	Description	Estimate	SE	RSE (%)	Estimate	95% CI
η1	ω^2^_V_	0.125 (CV = 36.5%)	0.0216	17.3% (Shr = 41.1%)	0.121	0.0675–0.364
η2	ω^2^_KA_	0.279 (CV = 56.7%)	0.0882	31.6% (Shr = 53.3%)	0.276	0.184–0.391
η3	ω^2^_CL_	0.138 (CV = 38.5%)	0.011	8% (Shr = 2.2%)	0.138	0.112–0.165
η4	ω^2^_ALAG1_	0.12 (CV = 35.7%)	0.0338	32.3% (Shr = 66.2%)	0.106	0.0439–0.27

Residual variability						
Epsilon	Description	Estimate	SE	RSE (%)	Estimate	95% CI
ε1	Proportional error	0.0468	0.0008	1.6% (Shr = 7.7%)	0.0463	0.0406–0.0523
ε2	Additive error	0.0938	0.0281	30% (Shr = 7.7%)	0.0864	0.0106–0.460

Note: SE, standard error; RSE, relative standard error; ALKPOT = 1 (no previous ALK inhibitor treatment), ALKPOT = 2, 3, and 4 (patients with previous ALK inhibitor treatment), ALKPOT = 5 (others (ROS1+ patients or unknown)), ALKPOT = 6 (healthy subjects).

**FIGURE 4:**
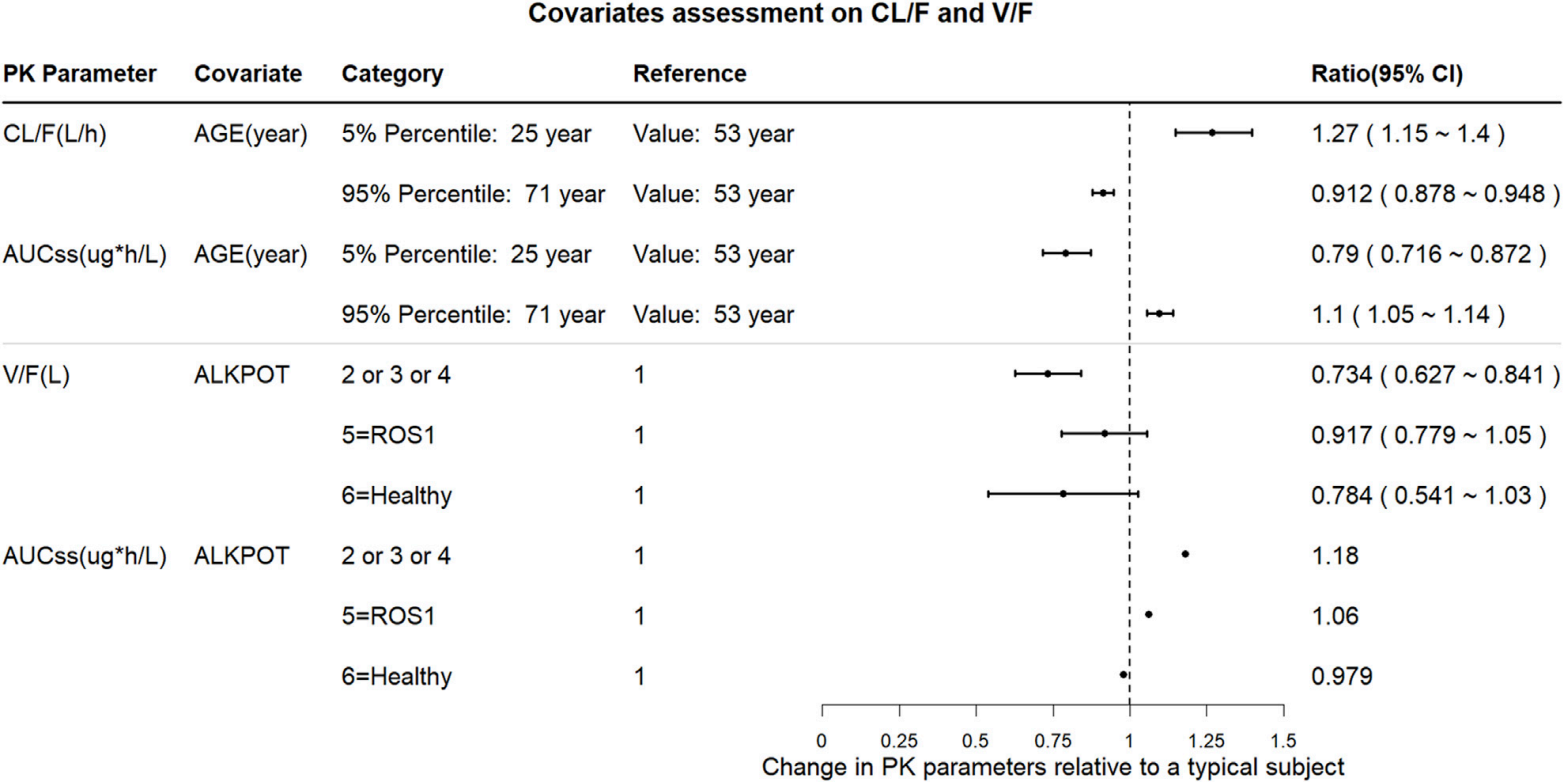
Forest plot of the influences of the significant covariates on SAF-189s steady-state exposure and PK parameters. The plots show the changes in steady-state PK parameters (CL/F, AUC_ss_, and V/F) relative to the reference group. The black solid circles are the changes in PK parameters for the covariate group compared with those in the corresponding reference group, and the horizontal lines represent the 90% prediction intervals (PIs) of change. ALKPOT = prior anticancer oncology therapy in ALK+ patients; 1 = no previous ALK inhibitor treatment; 2 = prior to enrolment, subjects were intolerable to only treatment with crizotinib; 3 = previous treatment with one medication of second- or third-generation of ALK inhibitors; 4 = previous treatment with at least two medications of second- or third-generation of ALK inhibitors; 5 = others (ROS1+ or unknown patients); 6 = healthy subjects. Compared with the median age (53 years), CL/F increased by 27% in the 5th percentile (25 years) and decreased by 8.8% in the 95th percentile (71 years), and AUC decreased by 21% and increased by 10% in the 5th and 95th percentiles, respectively. Prior anticancer therapy (ALKPOT = 2, 3, 4; ALKPOT = 5; and ALKPOT = 6) is a significant covariate for V/F. Compared to ALKPOT = 1, V/F is 26.6% lower when ALKPOT = 2, 3, 4; 8.3% lower when ALKPOT = 5; and 21.6% lower when ALKPOT = 6. Compared to ALKPOT = 1, AUC_ss_ is 18% higher when ALKPOT = 2, 3, 4; 6% higher when ALKPOT = 5; and 2.1% lower when ALKPOT = 6.

The impact of the PK covariates in Figure 4 was assessed using CL/F, V/F, and AUC_ss_ for the test conditions (5th and 95th percentiles of the continuous variables or test group of categorical variables) relative to the reference conditions. Compared with the median age (53 years), the CL/F increased by 27% in the 5th percentile (25 years) and decreased by 8.8% in the 95th percentile (71 years); the AUC decreased by 21% and increased by 10% in the 5th and 95th percentiles, respectively. Moreover, the covariates of prior anticancer therapy (ALKPOT = 2, 3, 4; ALKPOT = 5; and ALKPOT = 6) only had large effects on V/F because the point estimates of the change decreases of V/F were 0.734, 0.917, and 0.784, respectively, while the effect of prior anticancer therapy on AUC_ss_ was less than 20%. None of the other intrinsic factors (i.e., healthy vs. cancer patient, bodyweight, sex, preexisting mild hepatic impairment, and preexisting mild or moderate renal impairment) or extrinsic factors (i.e., concomitant medications) had clinically meaningful effects on SAF-189s systemic exposure.

### 3.2 Exposure-response analysis

**FIGURE 5:**
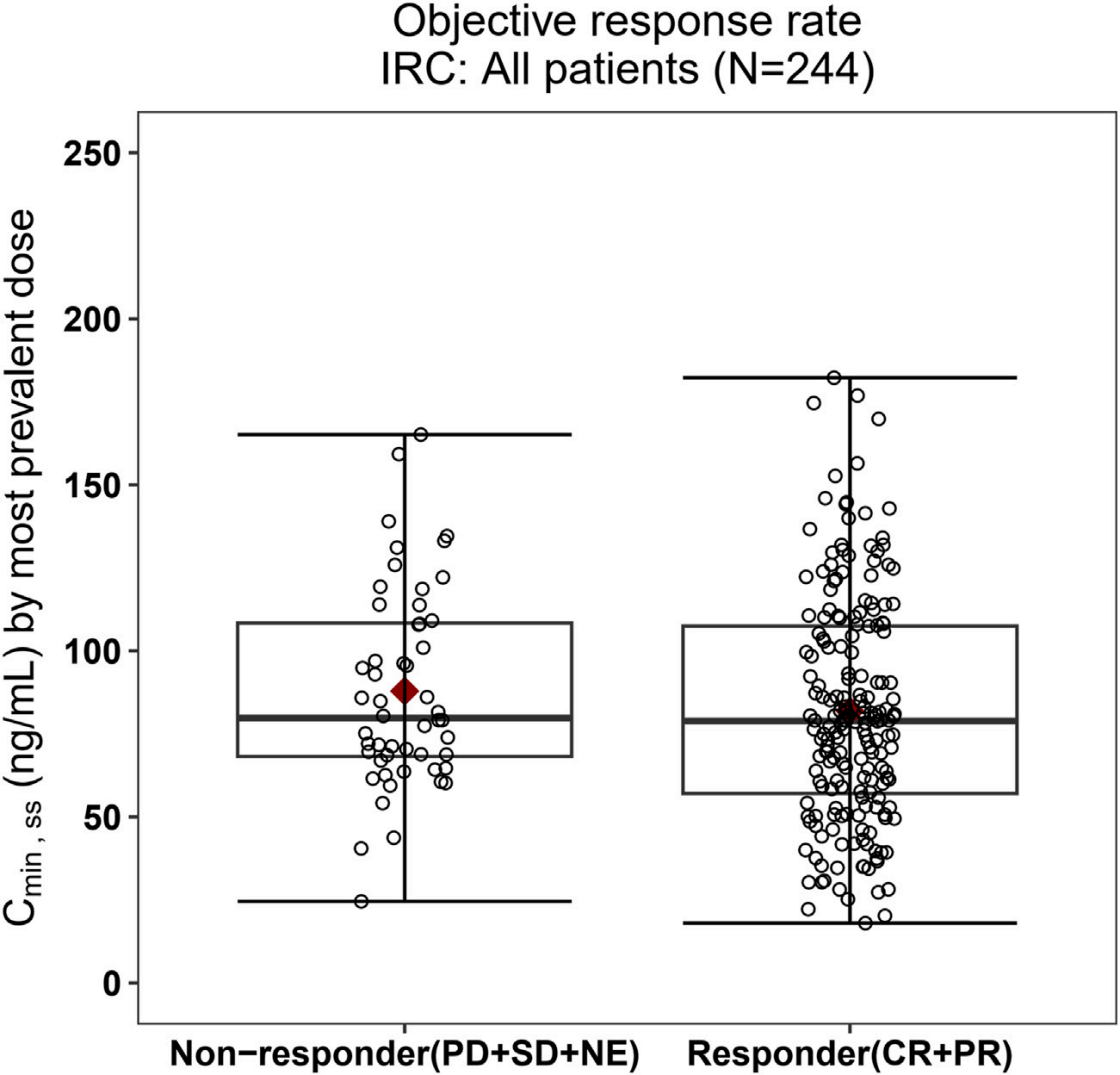
Boxplots of SAF-189s C_min,ss_ estimated using the most prevalent dose *versus* the overall response rate in all patients. The boxes display the 25th to 75th percentiles of C_min,ss_ in the responder and non-responder groups, and the whiskers represent 1.5 times the interquartile range; the black horizontal line within each box represents the median, and the red diamond represents the mean. There were no obvious difference in C_min,ss_ estimated using the most prevalent dose between the responders and non-responders.

**FIGURE 6:**
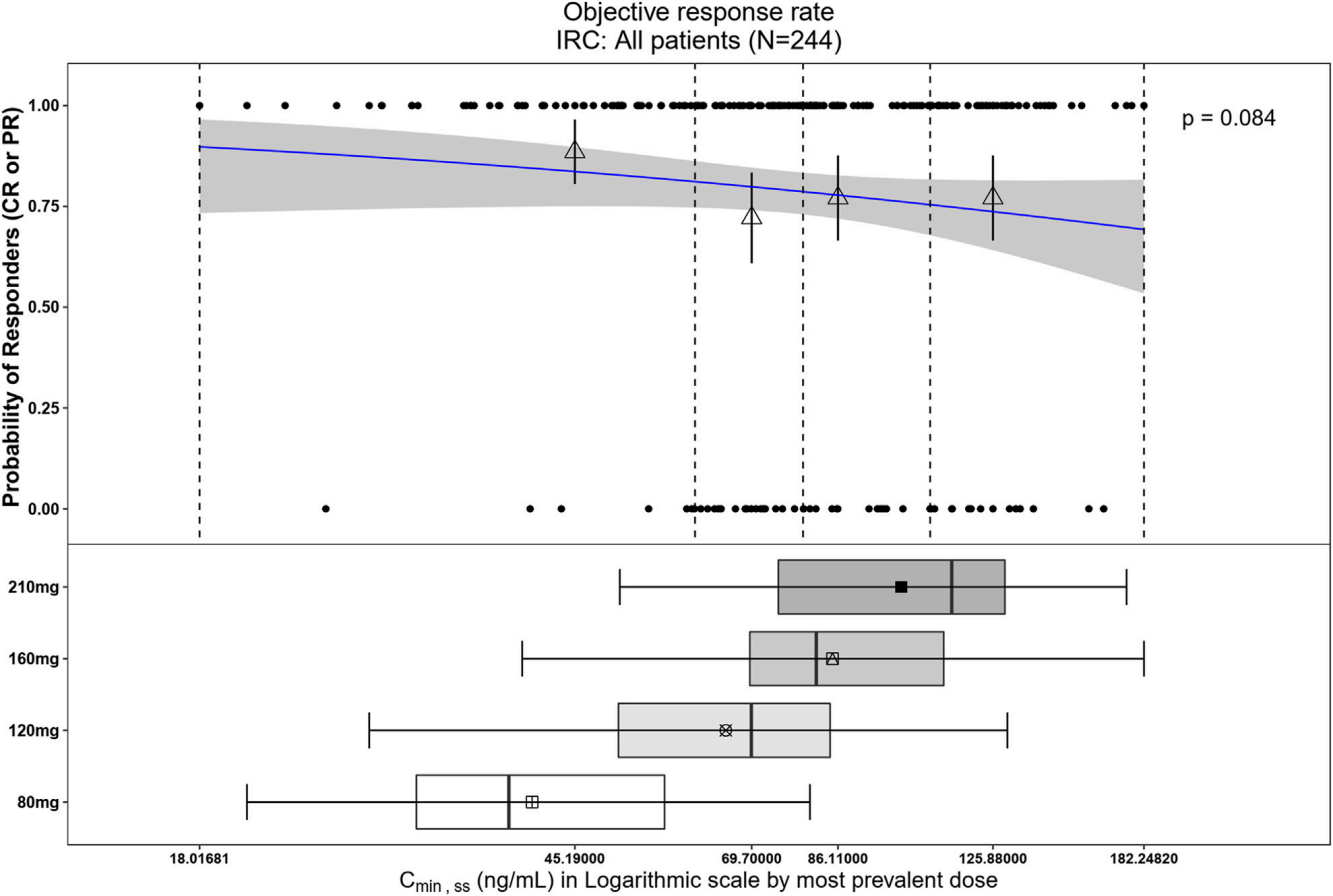
Observed data and model-predicted probability of responders in SAF-189s C_min,ss_ estimated using the most prevalent dose in all patients. In the upper portion of the figure, the blue solid line represents the fitted regression curve for the correlation between log(C_min,ss_) and probability of responders, the gray shading represents the 95% confidence interval, and the vertical black dotted line represents the C_min,ss_ quartiles: from left to right, 0% (lowest quartile), 25%, 50% (median), 75%, and 100% (largest quartile). The hollow triangles and whiskers represent the probability of responders in each of the four quartiles of C_min,ss_ and their 95% confidence intervals, respectively; for the filled circles in the y-axis direction, 0 represents non-responder and 1 represents responder patients, and the x-axis represents C_min,ss_ of the corresponding patients. The lower portion of the figure shows boxplots of C_min,ss_ for each of the dose groups. The results show no significant correlations between the probability of achieving ORR and C_min,ss_ across all subjects.

**FIGURE 7:**
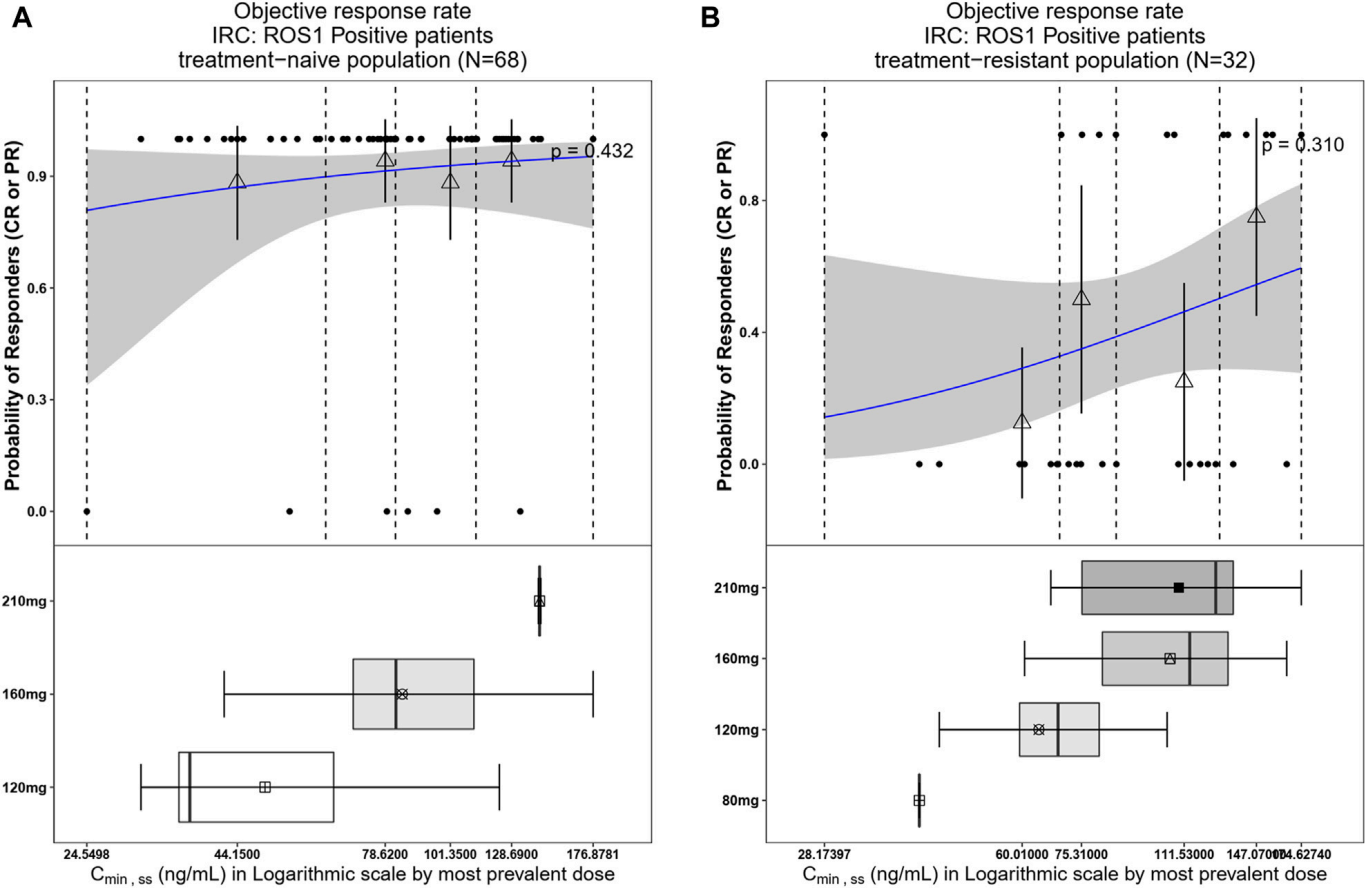
Observed data and model-predicted probability of responders in SAF-189s C_min,ss_ estimated using the most prevalent dose in ROS1+ patients. **(A)** Treatment-naïve ROS1+ patients; **(B)** treatment-resistant ROS1+ patients. In the upper portion of each figure, the blue solid lines represent the fitting regression curves of correlations between log(C_min,ss_) and probabilities of the responders, the gray shading represent the 95% confidence interval, and the vertical black dotted lines represent the C_min,ss_ quartiles: from left to right, 0% (lowest quartile), 25%, 50% (median), 75%, and 100% (largest quartile). The hollow triangles and whiskers represent the probability of responders in each of the four quartiles of C_min,ss_ and their 95% confidence intervals, respectively; for the filled circles in the y-axis direction, 0 represents non-responder and 1 represents responder patients, and the x-axis represents C_min,ss_ of the corresponding patients. The lower portion of each figure shows the boxplot of C_min,ss_ for each of the dose groups. The results show no significant correlations between the probability of achieving ORR and C_min,ss_ in both treatment-naïve and treatment-resistant ROS1+ subjects.

The relationship between exposure and efficacy was assessed based only on the phase II IRC assessment data (n = 244). For the ORR and PFS analyses, a total of 244 patients were included. In the DOR analysis, a total of 192 patients were included. C_min,ss_ and AUC_ss_ were chosen as the exposure parameters based on the correlation between exposure and efficacy as well as the E-R analysis results of the same target drug ceritinib (Lau et al., 2019). The C_min,ss_ estimates for the most prevalent dose for responders (CR + PR) and non-responders (PD + SD + NE) were similar and overlapping (Figure 5). There was no correlation between C_min,ss_ and probability of achieving clinical response (CR + PR) (Table 3). In addition, logistic regression analyses did not show a significant relationship between the probability of achieving ORR and C_min,ss_ in all patients (odds ratio (OR): 0.556; 95% CI: 0.285–1.083; *p* = 0.084) (Figure 6). The E-R relationship analysis was also conducted on the ROS1+ patients (stratified as treatment-naive and treatment-resistant populations), and the results were similar to those of the overall population, indicating no correlation between C_min,ss_ and the probability of achieving clinical response (CR + PR) (Figure 7). Exposure and efficacy analyses based on the AUC_ss_ showed a similar trend to that of C_min,ss_, and these results are provided in Supplementary document 2.

**TABLE 3:** Summary of the best overall responses stratified by SAF-189s C_min,ss_ quartiles estimated using the most prevalent dose.

C_min,ss_ quartile	C_min,ss_, ng mL^–1^	Number of patients	Incidence, N (%)				
			CR	PR	SD	PD	NE
1	18–60.7	61	1 (1.64)	53 (86.89)	4 (6.56)	3 (4.92)	0 (0.00)
2	60.7–79	61	0 (0.00)	44 (72.13)	13 (21.31)	4 (6.56)	0 (0.00)
3	79–108	61	0 (0.00)	47 (77.05)	12 (19.67)	2 (3.28)	0 (0.00)
4	108–182	61	1 (1.64)	46 (75.41)	11 (18.03)	3 (4.92)	0 (0.00)

**FIGURE 8:**
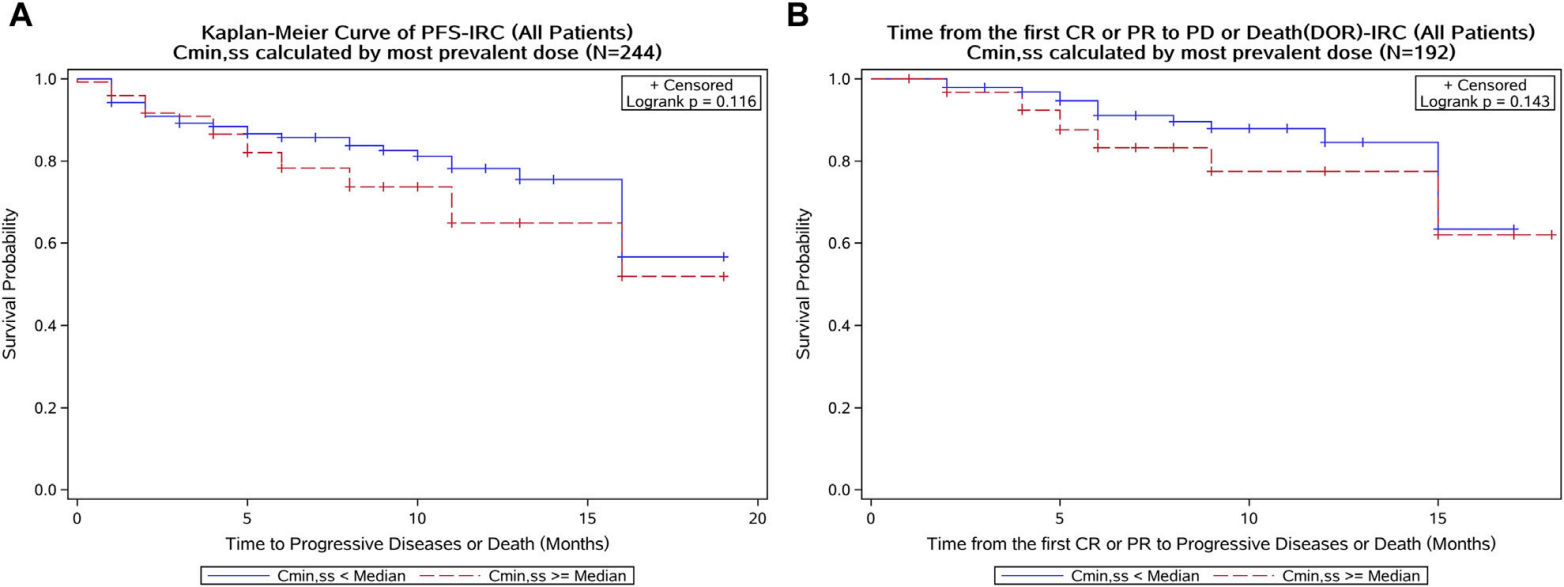
Kaplan–Meier curve of the PFS and DOR stratified by SAF-189s C_min,ss_ median estimated using the most prevalent dose in all patients. **(A)** Kaplan–Meier curve of PFS; **(B)** Kaplan–Meier curve of DOR. In each figure, the blue curve represents patients with C_min,ss_ below the median, while the red curve represents patients with C_min,ss_ above the median. A log-rank test was conducted to assess the difference in survival between these two groups. The *p*-value of the log-rank test was greater than 0.05, indicating no statistically significant differences in PFS and DOR between patients with lower and higher C_min,ss_ levels.

In the Kaplan–Meier analysis, no clear trend was displayed in the relationship between PFS/DOR and C_min,ss_ (Figure 8). Furthermore, the Cox regression model using C_min,ss_ as the continuous variable was tested for all patients, and the exposure parameters of the base Cox regression model were not significant predictors for PFS or DOR (*p* > 0.05) (Table 4). We also explored the relationship between exposure and tumor size through a tumor growth inhibition (SFBE) model, which indicated that the tumor size decreased rapidly after initiation of SAF-189s treatment in most patients; however, there was no trend observed between the exposure and tumor growth model parameters.

**TABLE 4:** Cox regression models for PFS and DOR in all patients.

Efficacy endpoint	Number of patients	Parameter	Estimate	SE	Hazard ratio (95% CI)	*p*-value
PFS	244	C_min,ss_	0.0071	0.0038	1.007 (1.000–1.015)	0.059
DOR	192	C_min,ss_	0.0059	0.0052	1.006 (0.996–1.016)	0.251

### 3.3 Exposure–safety analysis

**FIGURE 9:**
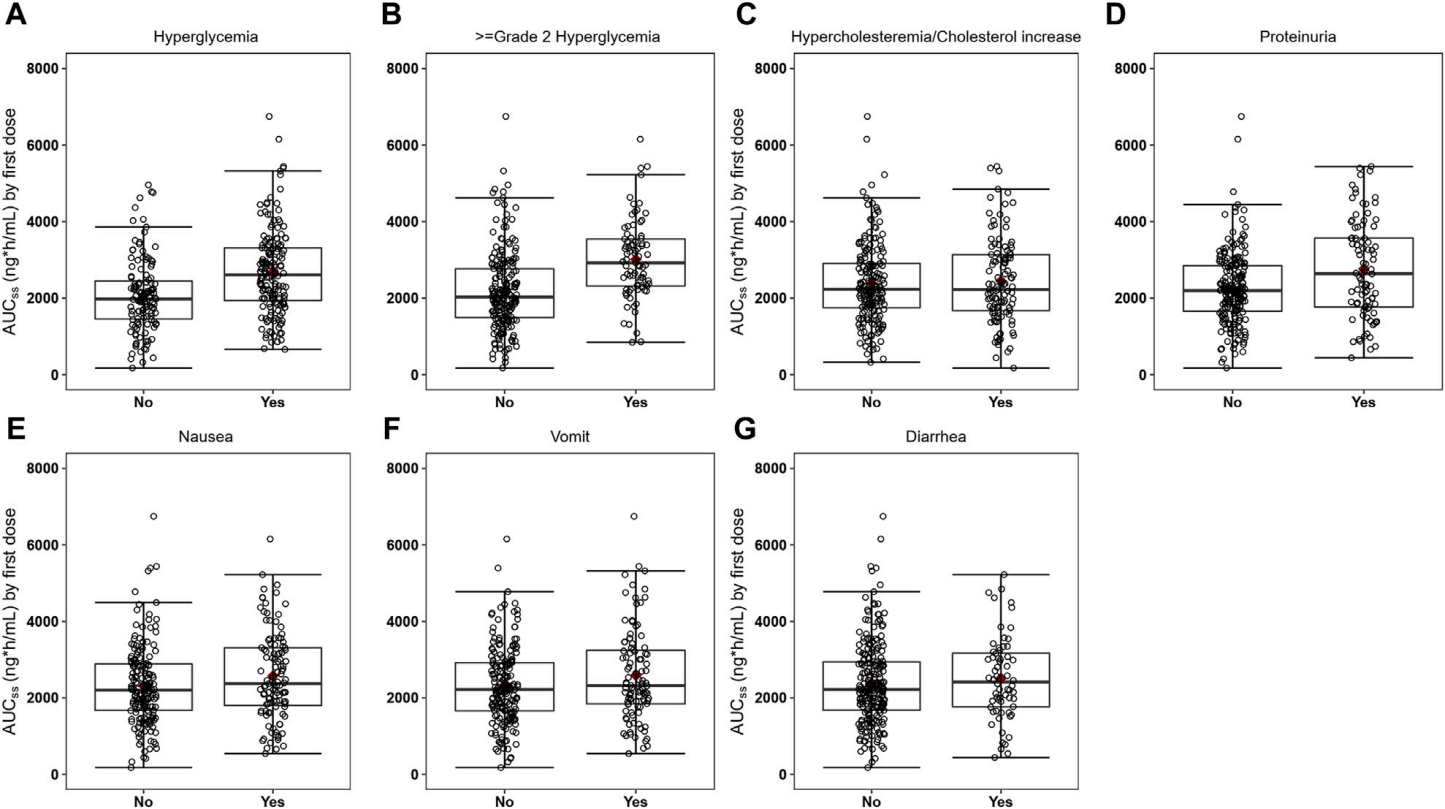
Boxplots of SAF-189s AUC_ss_ estimated from the first doses in patients with and without adverse events (AEs). Figures **(A–G)** represent hyperglycemia, grade ≥2 hyperglycemia, hypercholesterolemia/cholesterol increase, proteinuria, nausea, vomiting, and diarrhea. In each figure, the boxes display the 25th to 75th percentiles of AUC_ss_ in patients with and without AEs, whiskers represent 1.5 times the interquartile range, black horizontal lines within each of the boxes represent the median values, and red diamonds represent the mean values. Patients with any-grade or grade ≥2 hyperglycemia and any-grade proteinuria have median AUC_ss_ estimated by the first dose higher than those without these AEs. For the other safety endpoints, the exposure distributions were similar and overlapped among patients with and without each of the AEs.

In the analysis of relationship between exposure and incidence of AEs, 296 patients were included from the SAF001 study. The numbers of patients who experienced any-grade or grade ≥2 hyperglycemia, hypercholesterolemia or cholesterol increase, proteinuria, nausea, vomiting, and diarrhea are summarized in Table 5. AUC_ss_ was chosen as the exposure parameter because it reflects the overall exposure of the drug in the human body. Figure 9 shows that in patients with any-grade or grade ≥2 hyperglycemia and any-grade proteinuria, the median AUC_ss_ estimated by the first dose was higher than those without these AEs. For the other safety endpoints, the exposure distributions were similar and overlapped among the patients with or without each AE.

**TABLE 5:** Summary of numbers of patients with adverse events.

Adverse event	Number of patients (%)(N = 296)	
	Any grade	≥ Grade 2
Hyperglycemia	165 (55.74%)	82 (27.70%)
Hypercholesterolemia or cholesterol increase	111 (37.50%)	27 (9.12%)
Proteinuria	90 (30.41%)	28 (9.46%)
Nausea	112 (37.84%)	8 (2.70%)
Vomiting	102 (34.46%)	15 (5.07%)
Diarrhea	65 (21.96%)	17 (5.74%)

**FIGURE 10:**
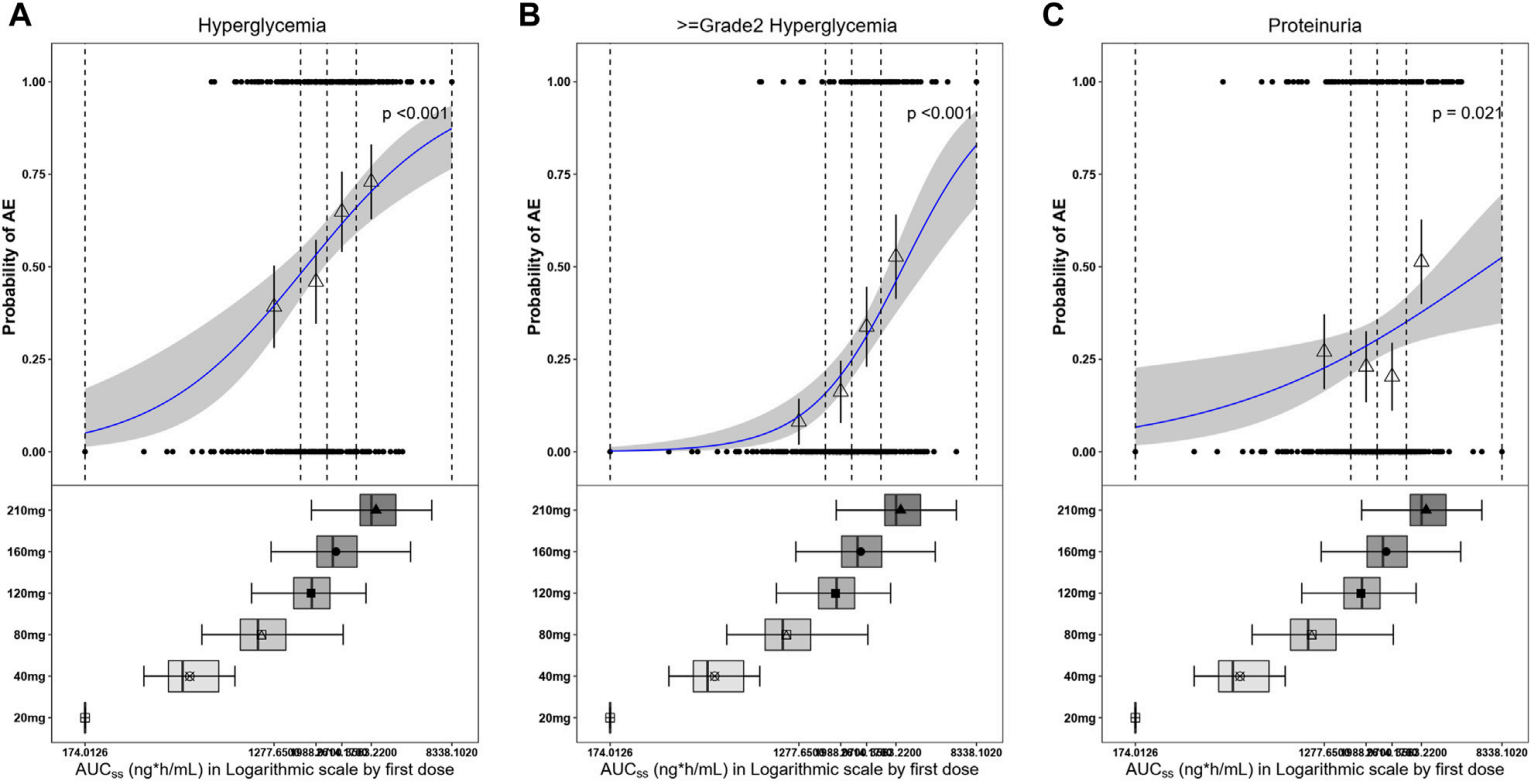
Observed and model-predicted probability of adverse events (AEs) *versus* SAF-189s AUC_ss_ estimated using the first dose. **(A)** Hyperglycemia; **(B)** grade ≥2 hyperglycemia; **(C)** proteinuria. In the upper portion of each figure, the blue solid line represents the fitting regression curve of correlation between log(AUC_ss_) and probability of a patient experiencing AE, the gray shading represents the 95% confidence interval, and the vertical black dotted line represents the AUC_ss_ quartiles: from left to right, 0% (lowest quartile), 25%, 50% (median), 75%, and 100% (largest quartile). The hollow triangles and whiskers represent the probabilities of patients experiencing AEs in each of the four quartiles of AUC_ss_ and their 95% confidence intervals, respectively; for the filled circles in the y-axis direction, 0 represents patients without AEs and 1 represents patients with AEs, and the x-axis represents AUC_ss_ of the corresponding patients. The lower portions of the figures show boxplots of AUC_ss_ for each of the dose groups. The results show that the probabilities of patients experiencing hyperglycemia, grade ≥2 hyperglycemia, and proteinuria increase with exposure.

Exposure–safety analyses were conducted using logistic regression, and a linear effect of log AUC_ss_ described the data well. The probability of a patient experiencing hyperglycemia, grade ≥2 hyperglycemia, and proteinuria would increase with exposure (Figure 10). For all patients, the ORs corresponding to an increase in SAF-189s exposure of 1 ng∙h mL^–1^ d^–1^ for any-grade hyperglycemia, grade ≥2 hyperglycemia, and proteinuria were 3.521 (95% CI: 2.103–5.897; *p* < 0.001), 7.662 (95% CI: 3.523–16.661, *p* < 0.001), and 2.031 (95% CI: 1.112–3.711, *p* = 0.021), respectively. The exposure and safety analyses based on C_min,ss_ and C_max,ss_ showed similar trends as that of the AUC_ss_, which is not presented herein. Moreover, by examining the boxplots of the exposure levels for each dosage group, it is evident that the incidence of AEs is notably higher in the high-dosage group. Furthermore, the 210-mg dosage group exhibited relatively inferior tolerability than the groups receiving lower doses.

## 4 Discussion

This is the first comprehensive PopPK and E-R analyses of SAF-189s. The final PopPK model of SAF-189s was described using a one-compartment model with delayed first-order absorption and time-dependent elimination by allowing the clearance to decrease stepwise over time. From the previous NCA results, a non-linear elimination trend was observed, with apparent clearance (CL/F) decline from 132 L h^–1^ after multiple doses at 20 mg once daily to 58.3 L h^–1^ at 210 mg. The *in vitro* studies revealed SAF-189s as a substrate and inhibitor of efflux transporter P-gp. Meanwhile, SAF-189s was also a substrate of CYP3A and could potentially induce non-linear time-dependent PK characteristics. Different models have been used to characterize the non-linear elimination of SAF-189s, including the non-linear Michaelis–Menten elimination, parallel linear and non-linear Michaelis–Menten elimination, and time-dependent elimination models, among which the time-dependent model with the best fit was selected finally (Lau et al., 2019). Several absorption models were tested, including first-order, zero-order, simultaneous zero-order and first-order, and parallel first-order absorption models with and without lag time. The absorption model with lag time has significantly lower OFVs and better GOFs than the models without lag time. Moreover, SAF-189s is a BCS IV drug, whose low solubility and low permeability may make the delayed absorption model more appropriate (Kratochwil et al., 2021). A more complex absorption model has no significant improvement on the GOF compared to simple first-order absorption. Therefore, first-order absorption with lag time was chosen to describe the absorption phase of the PK data of SAF-189s. In addition, there was no significant improvement in the GOF of the two-compartment model over the one-compartment model. Therefore, the one-compartment model was selected. In the final PopPK model, age was incorporated as a covariate for CL/F, while healthy subjects and prior anticancer therapy in ALK+ patients (ALKPOT) were incorporated for V/F. However, our analysis did not reveal an apparent E-R relationship for the ORR, PFS, or DOR under steady-state minimum concentration (C_min,ss_) and AUC_ss_. Therefore, although age and prior anticancer therapy significantly impact the PK parameters, they do not translate to clinically significant effects on SAF-189s exposure for the safety and efficacy outcomes. There were no apparent E-R relationships for the ORR, PFS, or DOR under steady-state minimum concentration or AUC_ss_ at doses ranging from 80 mg to 210 mg, although the PFS and DOR data were immature for patients participating in the phase II study at the time of this analysis. In addition, the same E-R relationship trends were observed in the ALK+ or ROS+ subgroups of subjects. The lack of an E-R relationship reveals that the observed flat E-R curve could potentially indicate that the exposures were in the plateau range of drug effects. Moreover, owing to the limited numbers of patients who were assigned dose levels above and below 160 mg QD and more overlaps in exposure between the 120-mg and 160-mg dose groups, the range of exposures studied did not permit adequate characterization of the lower part of the dose response curve.

The relationships between exposure and safety suggest that higher steady-state exposure may be associated with more frequent incidence of any-grade or grade ≥2 hyperglycemia and proteinuria, while there were no significant trends between exposure and incidence of other AEs, including hypercholesterolemia or cholesterol increase and gastrointestinal AEs (nausea, vomiting, and diarrhea). For SAF-189s, the incidence rate of hyperglycemia was relatively high (55.74%) compared with other ALK inhibitors. Based on current clinical use, crizotinib and alectinib had no effects on glucose tolerance in patients (Camidge et al., 2018; Seto et al., 2019; Wolf et al., 2022), while ceritinib dramatically induced hyperglycemia with an incidence rate of 49% in the FIH trial (Khozin et al., 2015). ALK is a member of the insulin-receptor protein tyrosine kinase superfamily (Kuo et al., 2007; Roskoski, 2013; Iragavarapu et al., 2015; Roskoski, 2017). The ATP-binding sites of ALK are similar to those of the insulin receptor (INSR) (Iragavarapu et al., 2015). According to the prescribing information of ceritinib, it blocks INSRs with greater potency than crizotinib (IC50: 7 vs. 290 nM) (Sakuma et al., 2019; Fujita et al., 2020). SAF-189s was developed on the basis of the structure of ceritinib with a lower IC50 of 0.8 nM, which would induce insulin resistance owing to its inhibitory effects on INSR.

The integrated pharmacometrics approach presented herein enables informed benefit–risk assessments for the use of SAF-189s in the treatment of ROS1-naive patients with NSCLC. The combined phase I and phase II data showed that SAF-189s has a large therapeutic window, with good efficacy in ALK+ and ROS1+ patients; the ORR for ALK+ and ROS1+ patients (n = 244) was 78.69% (95% CI: 73.92%–82.93%). In addition, there was no correlation between exposure and clinical response, and the efficacies of the 160-mg and 210-mg dose groups were comparable. The ROS1+ patients were also stratified as treatment-naive and treatment-resistant populations for the E-R relationship analysis; the results revealed no apparent correlations between exposure and efficacy in both populations. However, the analysis indicated better efficacy in the treatment-naive population. In the ROS1+ patients, the ORR of the treatment-resistant patients (n = 32) was 40.62% (95% CI: 23.70%–59.36%), while it was 91.18% (95% CI: 81.78%–96.69%) in the treatment-naive subgroup (n = 68). For the 160-mg dose group, the ORR of the ROS1+ treatment-naive patients (n = 59) was 89.83% (95% CI: 79.17%–96.18%). As subsequent studies progress and data become more abundant, further explorations of the E-R relationships will be conducted. The results of the exploratory analysis between safety and exposure show that when the exposure level AUC_ss_ was below the median value (2,233 ng∙h mL^–1^), the incidence of AEs (any grade or grade ≥2 hyperglycemia and proteinuria) was significantly less than that when the AUC_ss_ was above the median value. In this analysis, the geometric mean of the AUC_ss_ calculated at the 160 mg QD dose was 2,374 ng∙h mL^–1^, which was close to the median exposure value (2,233 ng∙h mL^–1^), indicating that 160 mg QD was well-tolerated with a manageable safety profile. The 210 mg dose was less tolerated than the other lower doses, with a significantly higher incidence of grade ≥3 treatment emergent adverse events (TEAE) and significantly higher incidence of TEAE leading to dose reduction. However, in the 160 mg dose group, the incidence of grade ≥3 TEAE was less than 9.15% in both the ALK+ and ROS1+ groups of patients (n = 153), and the incidence of drug-related TEAE leading to permanent discontinuation was low at 2%, indicating that the 160 mg QD dosing regimen was well tolerated. Based on the safety, efficacy, and PK data, 160 mg QD of SAF-189s was selected as the recommended dose in phase III.

While our study provides comprehensive analyses of the population pharmacokinetics and E-R relationships of SAF-189s, it has several limitations. First, the sample size, particularly for certain dose levels, was relatively small, thereby limiting the power to detect subtle E-R relationships. Second, the E-R data for PFS and DOR were immature at the time of analysis, potentially affecting the robustness of our conclusions. Third, the study population was restricted to Chinese patients, and these results may not be fully generalizable to other ethnic groups. Future studies with larger and more diverse populations as well as mature PFS and DOR data are needed to confirm and extend the findings. In this study, the patient age, health, and prior anticancer therapy were observed to play significant roles in explaining the IIV in SAF-189s PKs. However, the clinical significances of these covariates remain to be validated in a larger population.

## 5 Conclusion

The integrated pharmacometrics approach presented in this study helps with optimal benefit–risk assessments for the use of SAF-189s in the treatment of patients with ALK+/ROS1+ advanced NSCLC. No clinically significant covariates were identified in the proposed PopPK model. The results of the E-R relationships for the efficacy and safety endpoints within the dose range of 80–210 mg indicated that a dose of 160 mg QD had good balance between efficacy and tolerability. Thus, a dose of 160 mg QD for SAF-189s was selected as the recommended amount for phase III trials in ALK+/ROS1+ or ROS1+ NSCLC patients.

## Data Availability

The datasets presented in this article are not readily available because of the confidentiality of innovative antitumor drugs. This article contains the original contributions proposed in the study, and any further inquiries may be made to the corresponding authors.
