# Supplementary material for: Population pharmacokinetics and exposure–response analyses of SAF-189s in Chinese patients with ALK+/ROS1+ non-small cell lung cancer
Source: Front Pharmacol. 2024 Jul 16;15:1418549. doi: 10.3389/fphar.2024.1418549 (PMC11286589; doi:10.3389/fphar.2024.1418549)
Supplement: Supplementary file 1 [file Table1.docx]

Supplementary Material

**1 Supplementary document 1**

**Supplementary Table 1 Baseline Characteristics**

| **Continuous covariates,**  **median (range)** | **All patients**  **(N=317)** | **STL31147**  **(N=24)** | **SAF001-PHASEI**  **(N=45)** | **SAF001-PHASEII**  **(N=248)** |
| --- | --- | --- | --- | --- |
| Age, years | 51.6 (18.0-84.0) | 27 (18.0-45.0) | 51 (28.0-68.0) | 54.1 (20.0-84.0) |
| Albumin, g/L | 41.2 (23.0-52.6) | 48.2 (43.8-52.5) | 39.7 (24.8-50.9) | 40.8 (23.0-52.6) |
| Alkaline phosphatase, U/L | 109 (46.0-493) | 82.9 (54.0-142) | 120 (46.0-493) | 109 (46.0-448) |
| Alanine aminotransferase, U/L | 24.1 (2.00-127) | 32.2 (8.00-65.0) | 28 (6.00-99.0) | 22.6 (2.00-127) |
| Aspartate aminotransferase, U/L | 23.7 (9.00-106) | 22.9 (14.0-32.0) | 26.2 (13.0-52.9) | 23.3 (9.00-106) |
| Body mass index, kg/m^2^ | 23.7 (15.5-34.8) | 22.1 (19.2-25.8) | 23.1 (15.8-29.7) | 23.9 (15.5-34.8) |
| Blood urea nitrogen, mmol/L | 4.48 (1.03-9.40) | 3.97 (2.65-7.79) | 4.4 (1.90-9.17) | 4.55 (1.03-9.40) |
| Body weight, kg | 63.2 (37.3-92.5) | 65.5 (56.6-75.7) | 61.6 (39.0-90.5) | 63.3 (37.3-92.5) |
| Creatinine clearance, mL/min | 96.2 (48.8-199) | 121 (87.0-157) | 88.8 (48.8-129) | 95.1 (50.1-199) |
| Hemoglobin, g/L | 146 (20.0-210) | 160 (160-160) | 128 (20.0-210) | 149 (80.0-210) |
| Height, cm | 134 (90.5-181) | 151 (104-176) | 133 (100-164) | 132 (90.5-181) |
| Serum Creatinine, μmol/L | 163 (139-190) | 172 (161-186) | 163 (145-183) | 162 (139-190) |
| Total bilirubin, μmol/L | 68 (35.0-110) | 74.9 (61.0-91.0) | 72.3 (38.7-110) | 66.5 (35.0-108) |
| Total protein, g/L | 11.1 (2.00-32.2) | 12.9 (5.40-32.2) | 10.4 (2.00-26.4) | 11 (3.30-32.1) |
| eGFR, mL/min/1.73 m^2^ | 71.5 (44.6-91.0) | 76.5 (67.8-91.0) | 70.6 (55.8-83.3) | 71.2 (44.6-89.2) |
| **Categorical covariates,**  **n (%)** |  |  |  |  |
| Sex |  |  |  |  |
| Female | 153 (48.2) | 1 (4.2) | 19 (42.2) | 133 (53.6) |
| Male | 164 (51.8) | 23 (95.8) | 26 (57.8) | 115 (46.4) |
| Category of Age |  |  |  |  |
| Age<65 y | 272 (85.8) | 24 (100) | 41 (91.2) | 207 (83.4) |
| Age＞65 y | 45 (14.2) | 0 (0.00) | 4 (8.8) | 41 (16.6) |
| Smoking |  |  |  |  |
| Never | 201 (63.4) | 0 (0.00) | 28 (62.2) | 173 (69.8) |
| Current or Former | 116 (36.6) | 24 (100) | 17 (37.8) | 75 (30.2) |
| Alcohol consumption |  |  |  |  |
| Never | 245 (77.2) | 0 (0.00) | 38 (84.4) | 207 (83.4) |
| Current or Former | 72 (22.8) | 24 (100) | 7 (15.6) | 41 (16.6) |
| Fasting/Fed |  |  |  |  |
| Fasting | 317 (93) | 24 (50) | 45 (100) | 248 (100) |
| Fed | 24 (7) | 24 (50) | 0 (0.00) | 0 (0.00) |
| Concomitant medications |  |  |  |  |
| Absent | 172 (44.4) | 24 (100) | 34 (51.6) | 114 (38.4) |
| Present | 215 (55.6) | 0 (0.00) | 32 (48.4) | 183 (61.6) |
| coadministration with metformin |  |  |  |  |
| Absent | 293 (86.4) | 24 (100) | 43 (95.6) | 226 (83.8) |
| Present | 46 (13.6) | 0 (0.00) | 2 (4.4) | 44 (16.2) |
| Medical history |  |  |  |  |
| Absent | 43 (13.6) | 24 (100) | 3 (6.6) | 16 (6.4) |
| Present | 274 (86.4) | 0 (0.00) | 42 (93.4) | 232 (93.6) |
| Hepatic function |  |  |  |  |
| Normal | 276 (87) | 23 (95.8) | 34 (75.6) | 219 (88.4) |
| Mild Dysfunction | 41 (13) | 1 (4.2) | 11 (24.4) | 29 (11.6) |
| Renal function |  |  |  |  |
| Normal | 207 (65.2) | 17 (70.8) | 27 (60) | 163 (65.8) |
| Mild Dysfunction | 94 (29.6) | 0 (0.00) | 18 (40) | 76 (30.6) |
| Moderate Dysfunction | 1 (0.4) | 0 (0.00) | 0 (0.00) | 1 (0.4) |
| Other(130≤eGFR) | 15 (4.8) | 7 (29.2) | 0 (0.00) | 8 (3.2) |
| ECOG |  |  |  |  |
| 0 | 80 (25.2) | 24 (100) | 8 (17.8) | 48 (19.4) |
| 1 | 226 (71.2) | 0 (0.00) | 37 (82.2) | 189 (76.2) |
| 2 | 11 (3.4) | 0 (0.00) | 0 (0.00) | 11 (4.4) |
| Prior Anti-cancer Therapy in ROS1+ patients |  |  |  |  |
| 1 | 71 (22.4) | 0 (0.00) | 0 (0.00) | 71 (28.6) |
| 2 | 26 (8.2) | 0 (0.00) | 0 (0.00) | 26 (10.4) |
| 3 | 6 (1.8) | 0 (0.00) | 0 (0.00) | 6 (2.4) |
| 4 | 214 (67.6) | 24 (100) | 45 (100) | 145 (58.4) |
| Prior Anti-cancer Therapy in ALK+ patients |  |  |  |  |
| 1 | 115 (36.2) | 0 (0.00) | 11 (24.4) | 104 (42) |
| 2 | 45 (14.2) | 0 (0.00) | 22 (48.8) | 23 (9.2) |
| 3 | 10 (3.2) | 0 (0.00) | 0 (0.00) | 10 (4) |
| 4 | 9 (2.8) | 0 (0.00) | 1 (2.2) | 8 (3.2) |
| 5 | 114 (36) | 0 (0) | 11 (24.4) | 103 (41.6) |
| 6 | 24(7.6) | 24(100) | 0 (0.00) | 0 (0.00) |
| Brain metastases at enrollment |  |  |  |  |
| Absent | 181 (57) | 24 (100) | 18 (40) | 139 (56) |
| Present | 136 (43) | 0 (0.00) | 27 (60) | 109 (44) |
| Disease stage |  |  |  |  |
| I | 3 (1) | 0 (0.00) | 1 (2.2) | 2 (0.8) |
| II | 10 (3.2) | 0 (0.00) | 1 (2.2) | 9 (3.6) |
| III | 50 (15.8) | 0 (0.00) | 12 (26.6) | 38 (15.4) |
| IV | 221 (69.8) | 0 (0.00) | 30 (66.6) | 191 (77) |
| Other | 33 (10.4) | 24 (100) | 1 (2.2) | 8 (3.2) |
| ALK Mutation |  |  |  |  |
| Positive | 190 (60) | 0 (0.00) | 45 (100) | 145 (58.4) |
| Negative or Other | 127 (40) | 24 (100) | 0 (0.00) | 103 (41.6) |
| ROS1 Mutation |  |  |  |  |
| Positive | 103 (32.4) | 0 (0.00) | 0 (0.00) | 103 (41.6) |
| Negative or Other | 214 (67.6) | 24 (100) | 45 (100) | 145 (58.4) |
| Subject Type |  |  |  |  |
| Healthy | 24 (7.6) | 24 (100) | 0 (0.00) | 0 (0.00) |
| ALK+ only patients | 190 (60) | 0 (0.00) | 45 (100) | 145 (58.4) |
| ROS1+ only patients | 103 (32.4) | 0 (0.00) | 0 (0.00) | 103 (41.6) |
| Dosage of SAF-189s |  |  |  |  |
| 20 mg | 2 (0.6) | 0 (0.00) | 2 (4.4) | 0 (0.00) |
| 40 mg | 6 (1.8) | 0 (0.00) | 6 (13.4) | 0 (0.00) |
| 80mg | 28 (8.8) | 0 (0.00) | 8 (17.8) | 20 (8) |
| 120 mg | 71 (22.4) | 0 (0.00) | 9 (20) | 62 (25) |
| 160 mg | 174 (54.8) | 24 (100) | 9 (20) | 141 (56.8) |
| 210 mg | 36 (11.4) | 0 (0.00) | 11 (24.4) | 25 (10) |

Note: For Prior Anti-cancer Therapy in ROS1+ patients, 1 = No previous ROS1 inhibitor treatment; 2 = Prior to enrollment, subjects were intolerable to the only treatment with crizotinib;3 = other situations in ROS1+ patients; 4 = Others (Healthy or ALK+ patients) or Unknown ; For Prior Anti-cancer Therapy in ALK+ patients, 1= No previous ALK inhibitor treatment; 2= Prior to enrollment, subjects were intolerable to the only treatment with crizotinib; 3=Previous treatment with one medication of 2nd- or 3rd-generation of ALK inhibitors; 4=Previous treatment with at least two medications of 2nd- or 3rd-generation of ALK inhibitors; 5= Others (ROS1+ or Unknown patients); 6= Healthy subjects.
