# Supplementary material for: Population pharmacokinetics and exposure–response analyses of SAF-189s in Chinese patients with ALK+/ROS1+ non-small cell lung cancer
Source: Front Pharmacol. 2024 Jul 16;15:1418549. doi: 10.3389/fphar.2024.1418549 (PMC11286589; doi:10.3389/fphar.2024.1418549)
Supplement: Supplementary file 2 [file DataSheet1.docx]

Supplementary Material

**Supplementary document 2**


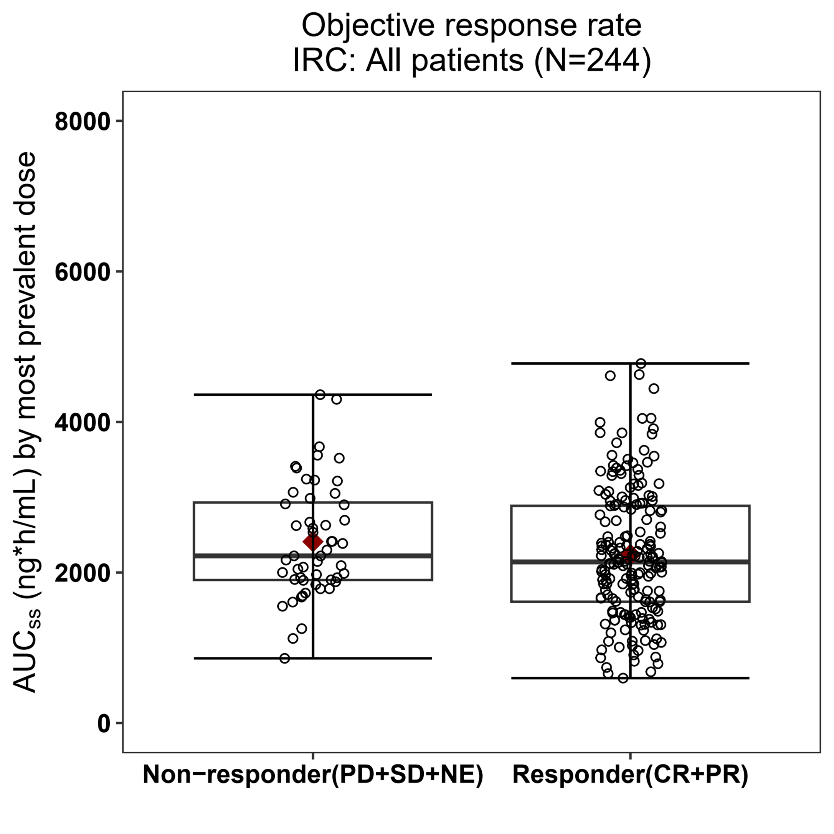


Figure 1 Box plots of SAF-189s AUC_ss_ estimated by the most prevalent dose versus overall response rate in all patients. Boxes display the 25th to 75th percentiles of AUC_ss_ in responder and non-responder, whiskers represent representing 1.5 times the interquartile range, the black horizontal line within each box represent the median, the red diamond represents the mean. There was no obvious difference in AUC_ss_ estimated by the most prevalent dose between responders and non-responders.


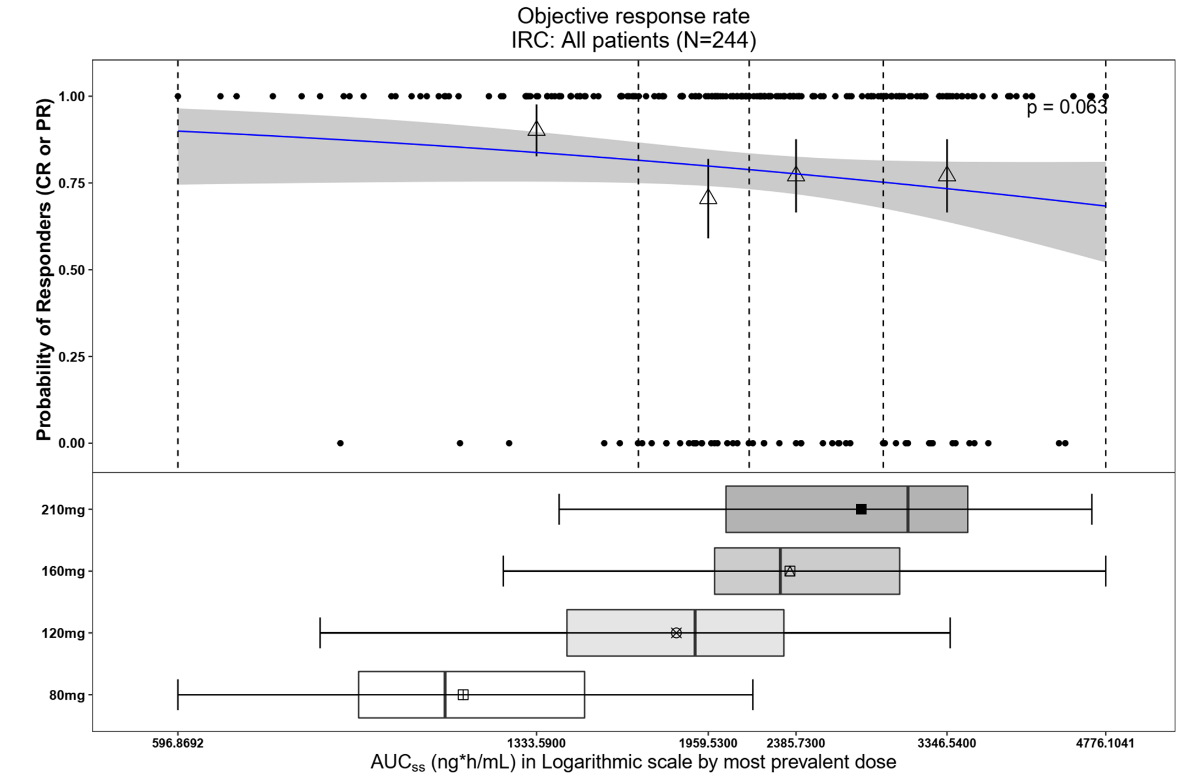


Figure 2 Observed data and model predicted probability of responders in SAF-189s AUC_ss_ estimated by the most prevalent dose in all patients. In the upper portion of the figure. The blue solid line represents the fitting regression curve of correlation between log(AUC_ss_) and probability of responders, the grey shadow represents the 95% confidence interval, the vertical black dotted line represents the AUC_ss_ quartiles: from left to right: 0% (lowest quartile), 25%, 50% (median), 75% and 100% (largest quartile); the hollow triangle and its whiskers represent the probability of responders in each four quartiles of AUC_ss_ and its 95% confidence interval; The filled circle in the y-axis= 0 represents non-responders patients; the filled circle on y-axis=1 represents responders and its x-axis represents AUC_ss_ of the corresponding patient. The lower portion of the figure shows box plot of AUC_ss_ for each dose group. The results show no significant correlation between the probability of achieving ORR and AUC_ss_ across all subjects.


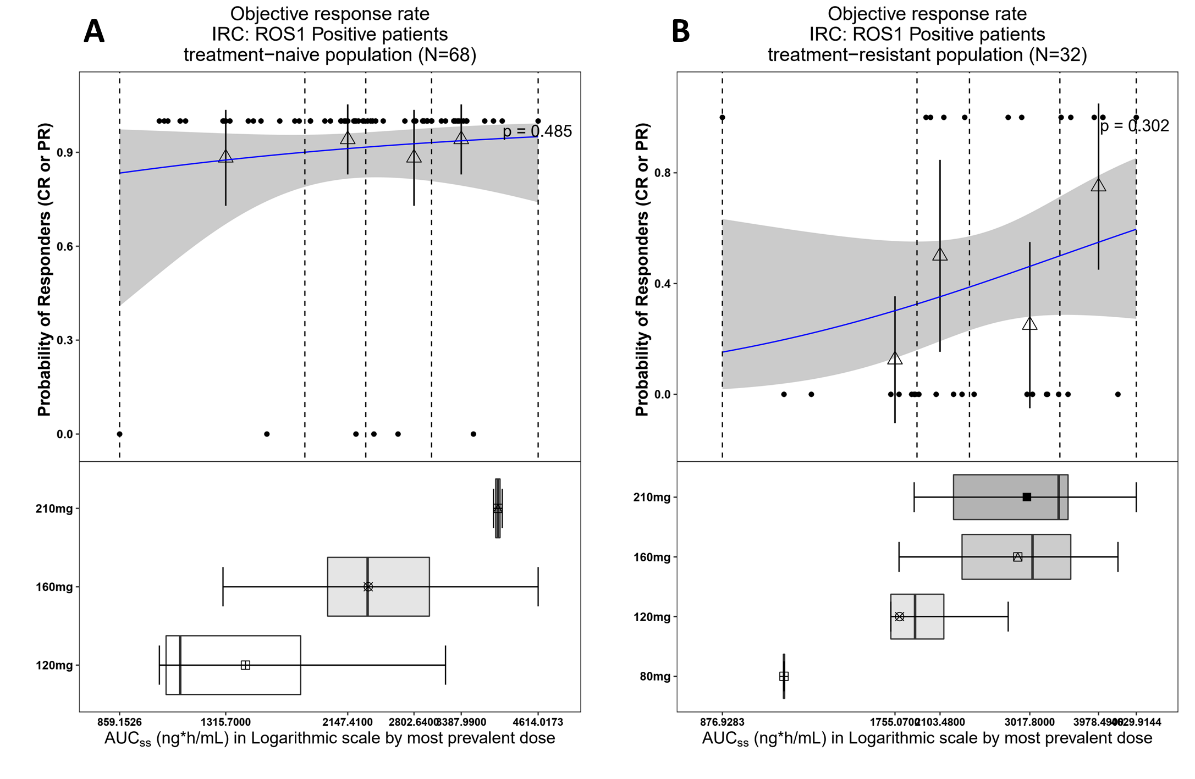


Figure 3 Observed data and model predicted probability of responders in SAF-189s AUC_ss_ estimated by the most prevalent dose in ROS1+ patients. (A) Treatment-naïve ROS1+ patients; (B) Treatment- resistant ROS1+ patients. In the upper portion of each figure, the blue solid line represents the fitting regression curve of correlation between log(AUC_ss_) and probability of responders, the grey shadow represents the 95% confidence interval, the vertical black dotted line represents the AUC_ss_ quartiles: from left to right: 0% (lowest quartile), 25%, 50% (median), 75% and 100% (largest quartile); the hollow triangle and its whiskers represent the probability of responders in each four quartiles of AUC_ss_ and its 95% confidence interval; The filled circle in the y-axis= 0 represents non-responders patients; the filled circle on y-axis=1 represents responders and its x-axis represents AUC_ss_ of the corresponding patient. The lower portion of each figure shows box plot of AUC_ss_ for each dose group. The results show no significant correlation between the probability of achieving ORR and AUC_ss_ in both treatment-naïve and treatment- resistant ROS1+ subjects.


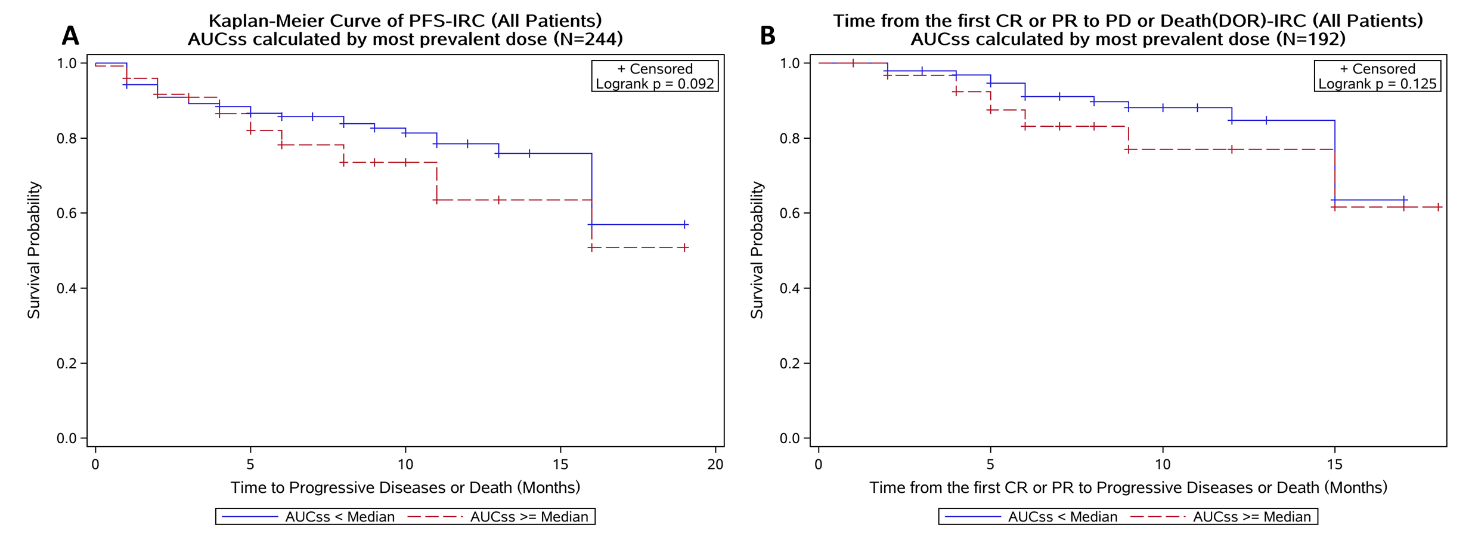


Figure 4 Kaplan-Meier curve of PFS and DOR, stratified by SAF-189s AUC_ss_ median estimated by the most prevalent dose in all patients. (A) Kaplan-Meier curve of PFS; (B) Kaplan-Meier curve of DOR. In each figure, the blue curve represents patients with AUC_ss_ below the median, while the red curve represents patients with AUC_ss_ above the median. A log-rank test was conducted to assess the difference in survival between these two groups. The p-value of the log-rank test was greater than 0.05, indicating no statistically significant difference in PFS and DOR between patients with lower and higher AUC_ss_ levels.
